# Spontaneous, local diastolic subsarcolemmal calcium releases in single, isolated guinea-pig sinoatrial nodal cells

**DOI:** 10.1371/journal.pone.0185222

**Published:** 2017-09-25

**Authors:** Syevda G. Sirenko, Dongmei Yang, Larissa A. Maltseva, Mary S. Kim, Edward G. Lakatta, Victor A. Maltsev

**Affiliations:** Laboratory of Cardiovascular Science, National Institute on Aging, National Institutes of Health, Baltimore, Maryland, United States of America; Indiana University School of Medicine, UNITED STATES

## Abstract

Uptake and release calcium from the sarcoplasmic reticulum (SR) (dubbed “calcium clock”), in the form of spontaneous, rhythmic, local diastolic calcium releases (LCRs), together with voltage-sensitive ion channels (membrane clock) form a coupled system that regulates the action potential (AP) firing rate. LCRs activate Sodium/Calcium exchanger (NCX) that accelerates diastolic depolarization and thus participating in regulation of the time at which the next AP will occur. Previous studies in rabbit SA node cells (SANC) demonstrated that the basal AP cycle length (APCL) is tightly coupled to the basal LCR period (time from the prior AP-induced Ca^2+^ transient to the diastolic LCR occurrence), and that this coupling is further modulated by autonomic receptor stimulation. Although spontaneous LCRs during diastolic depolarization have been reported in SANC of various species (rabbit, cat, mouse, toad), prior studies have failed to detect LCRs in spontaneously beating SANC of guinea-pig, a species that has been traditionally used in studies of cardiac pacemaker cell function. We performed a detailed investigation of whether guinea-pig SANC generate LCRs and whether they play a similar key role in regulation of the AP firing rate. We used two different approaches, 2D high-speed camera and classical line-scan confocal imaging. Positioning the scan-line beneath sarcolemma, parallel to the long axis of the cell, we found that rhythmically beating guinea-pig SANC do, indeed, generate spontaneous, diastolic LCRs beneath the surface membrane. The average key LCR characteristics measured in confocal images in guinea-pig SANC were comparable to rabbit SANC, both in the basal state and in the presence of β-adrenergic receptor stimulation. Moreover, the relationship between the LCR period and APCL was subtended by the same linear function. Thus, LCRs in guinea-pig SANC contribute to the diastolic depolarization and APCL regulation. Our findings indicate that coupled-clock system regulation of APCL is a general, species-independent, mechanism of pacemaker cell normal automaticity. Lack of LCRs in prior studies is likely explained by technical issues, as individual LCRs are small stochastic events occurring mainly near the cell border.

## Introduction

The importance of calcium (Ca^2+^) for cardiac pacemaker cell function has been established long ago mainly via pharmacological interventions targeting sarcoplasmic reticulum (SR) Ca^2+^ cycling [1–6] (for review see [7]). More recent studies have established specific mechanisms on how Ca^2+^ contributes to the regulation of action potential (AP) firing rate: SR (dubbed Ca^2+^ clock) generates spontaneous diastolic local calcium releases (LCRs) that activate Sodium/Calcium exchanger (NCX) inward current accelerating the diastolic depolarization [8, 9]. Normal automaticity of rabbit SANC depends upon efficiency of the coupling between the Ca^2+^ clock that generates LCRs and the ensemble of sarcolemmal electrogenic molecules (dubbed membrane clock), forming a coupled-clock system [10, 11]. The rhythmic LCRs generation is driven by a high basal level of phosphorylation of Ca^2+^ cycling proteins in rabbit SA node cells (SANC) [12]. Single rabbit SANC have documented tight correlations between the LCR period (time from the peak of the prior AP to the onset of LCR in diastole) and action potential cycle length (APCL), both in basal state [13], and during its modulation by PKA-dependent phosphorylation in response to autonomic receptor stimulation [12, 14, 15].

Spontaneous, diastolic LCRs have been reported in species other than rabbit and in different pacemaker cell types: mouse SANC [16], cat latent atrial pacemaker cells [9], toad pacemaker cells [1], mouse stem-cell derived cardiac cells [17, 18]. Thus, such LCRs appear to be a general feature of cardiac pacemaker cells. However, previous studies [19] did not detect LCRs in guinea-pig SANC, which have been widely employed in studies of pacemaker cell function. Here we addressed the issue of whether the guinea-pig SANC generate LCRs and whether LCRs participate in regulation of pacemaker function. We employed confocal line-scan imaging to detect and measure LCRs in guinea-pig SANC. We also performed parallel studies in rabbit SANC in order to compare LCR characteristics in both species. Ca^2+^ measurements were also performed using a 2D high-speed camera to detect LCRs not only along a scan-line but also within the entire cell perimeter. Our measurements demonstrated that guinea-pig SANC do indeed generate spontaneous, diastolic LCRs. Our results also confirmed that relationship between the LCR period and APCL in rabbit SANC remains valid in the basal state and in the presence of β-Adrenergic Receptor stimulation (BARs) in guinea-pig SANC. Thus, our results support the idea that the coupled-clock system [10] is a general mechanism of cardiac pacemaker cell function.

## Materials and methods

### Ethics statement

The present study conformed to the Guide for the Care and Use of Laboratory Animals, published by the US National Institutes of Health. The experimental protocols were approved by the Animal Care and Use Committee of the National Institutes of Health (protocol # # 034-LCS-2019). New Zealand White rabbits (Charles River Laboratories, USA) weighing 2.8–3.2 Kg were deeply anesthetized with sodium pentobarbital (50–90 mg/kg) injected to the central ear vein. The adequacy of anesthesia was monitored in rabbits until reflexes to ear and tale pinch and jaw tone were lost. The guinea pigs (Charles River Laboratories, USA) weighed 500–650 grams and were acutely anesthetized with pentobarbital sodium using IP injection at approximately 150 mg/kg doses to an absence of toe pinch reflex and eye membrane retraction.

### SANC preparations

The present study used isolated single SANC from guinea-pig and rabbit as previously described for rabbit SANC [20]. The heart was removed quickly and placed in the solution containing (in mmol/L): 130 NaCl, 24 NaHCO_3_, 1.2 NaH_2_PO_4_, 1.0 MgCl_2_, 1.8 CaCl_2_, 4.0 KCl, 5.6 glucose equilibrated with 95% O_2_ / 5% CO_2_ (pH 7.4 at 35.5°C). The sinoatrial node (SA) region was cut into small strips (~1.0 mm wide) perpendicular to the crista terminalis and excised. The final SA node preparation consisted of SA node strips attached to the small portion of crista terminalis. The SA node preparation was washed twice in Ca^2+^-free solution containing (in mmol/L): 140 NaCl, 5.4 KCl, 0.5 MgCl2, 0.33 NaH_2_PO_4_, 5 HEPES, 5.5 glucose, (pH = 6.9) and incubated at 35.5°C for 30 min in the same solution with addition of elastase type IIA (0.6 mg/ml; Sigma, Chemical Co.), collagenase type 2 (0.6 mg/ml; Worthington, NJ, USA) and 0.1% bovine serum albumin (Sigma, Chemical Co.). The SA node preparation was washed in modified Kraftbruhe (KB) solution, containing (in mmol/L): 70 potassium glutamate, 30 KCl, 10 KH_2_PO_4_, 1 MgCl_2_, 20 taurine, 10 glucose, 0.3 EGTA, and 10 HEPES (titrated to pH 7.4 with KOH), and kept at 4°C for 1h in KB solution containing 50 mg/ml polyvinylpyrrolidone (PVP, Sigma, Chemical Co.). Finally, cells were dispersed from the SA node preparation by gentle pipetting in the KB solution and stored at 4°C.

### Confocal line-scan imaging of AP-induced Ca^2+^ transients and spontaneous diastolic LCRs

Ca^2+^ transients and spontaneous diastolic LCRs were recorded in intact rhythmically beating guinea-pig and rabbit SANC by a LSM510 confocal microscope (Carl Zeiss, Germany) using a 40X1.3 oil objective. We used a 488nm argon ion laser with intensity attenuated to 1–3% for excitation of Fluo-4, and fluorescence emission was collected at wavelength of >505 nm. All images were recorded in the line-scan mode at a rate 1.92 or 3 ms per scan-line.

SANC were loaded with fluo-4 AM (5–10 μmol/L) for 10–15 min. After loading, cells were washed in Tyrode’s solution containing in mmol/L: 140 NaCl, 5.4 KCl, 5 HEPES, 2 MgCl_2_, 1.8 CaCl_2_, 10 glucose, (pH 7.4). Ca^2+^ transients and spontaneous diastolic local Ca^2+^ releases (LCRs) were recorded in both cell types superfused with Tyrode’s solution (above) at baseline and during β-adrenergic receptor stimulation, BARs (1 μM Isoproterenol, ISO) at 35±0.1°C. The line-scan in all experiments was oriented along the long axis of the SANC close to the sarcolemmal membrane.

**Fig 1:**
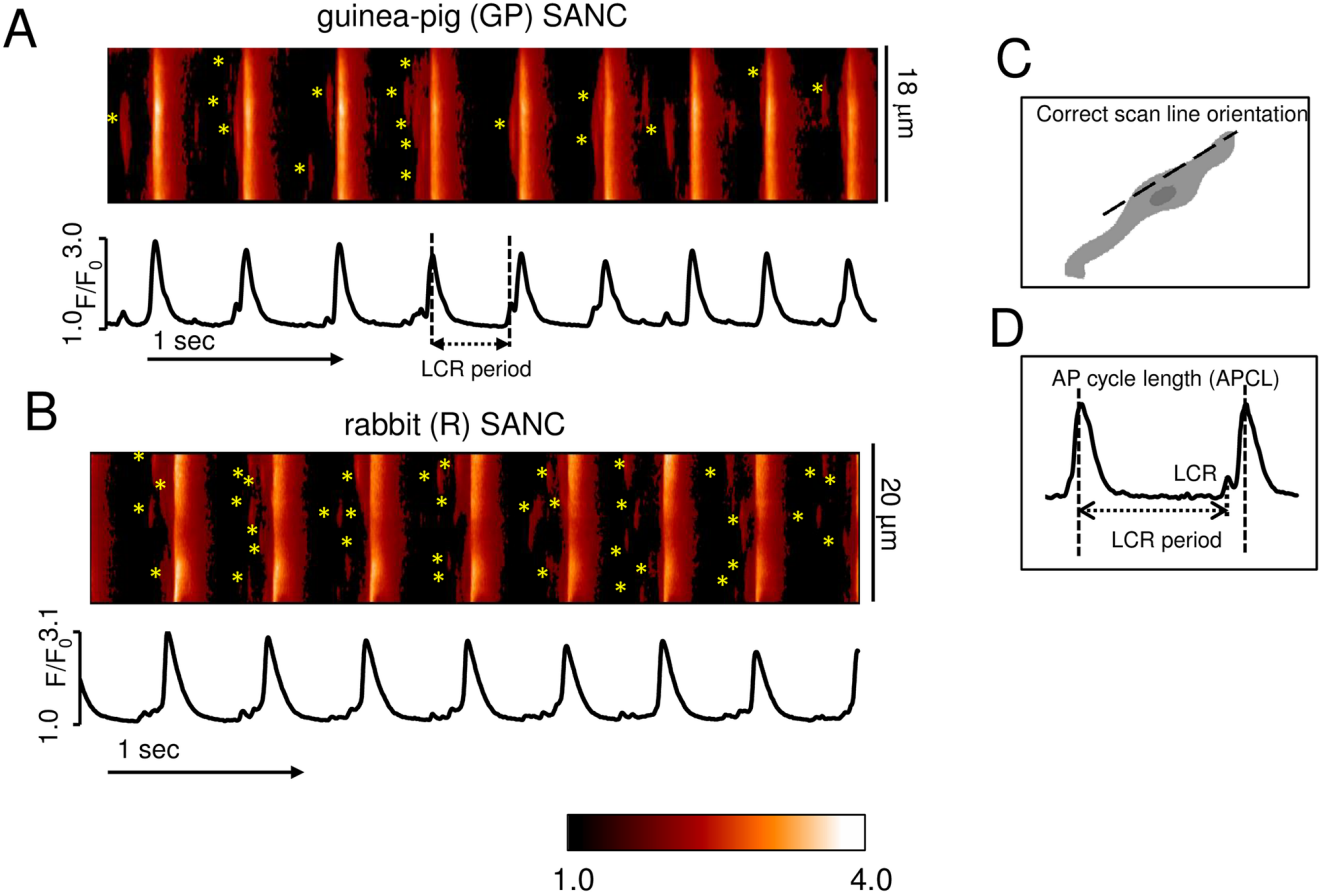
Both GP and R single rhythmically beating SANC generate spontaneous diastolic LCRs beneath sarcolemma under the basal conditions. Representative examples of confocal line scan images of LCRs (marked with asterisks) and AP-induced Ca^2+^ transient recorded in SANC of GP (A) and R (B). (C) Schematic illustration of the correct scan-line orientation along the cell border. (D) Definition of the LCR period and AP cycle length (APCL).

We took care to set the scan-line specifically along the border of the cell. An additional important consideration of setting the scan-line is that not every location beneath the sarcolemma generates LCRs each cycle [13]. Thus, to detect LCRs, the scanning line should not be set randomly, but rather within specific cell locations of LCR preferable activity over several cycles determined by prescreening with x-y confocal imaging (Fig 1C and S1 Fig). Imaging contrast and balanced laser power for Ca^2+^ indicator excitation are other crucial factors in successful detection of LCRs. Loading SANC with fluorescent probes to an optimal degree that generate robust Ca^2+^ signal with minimal buffering is critical. The average beating rates of unloaded and loaded with fluo-4AM cells were similar (2.8±0.23 Hz, n = 8 and 2.8±0.26 Hz, n = 13 respectively).

### Analysis of diastolic LCRs recorded by confocal microscopy

Fluo-4 measurements of LCRs in intact SANC are presented as F/F_0_ (normalized Ca^2+^ signal). LCR spatial size (μm) was indexed as the full width at the half maximum amplitude (FWHM), and its duration (ms) characterized as the full duration at half maximum amplitude (FDHM). Because the spatial extend (length) and duration of scanning varied in each experiment, we report the average LCR rate (frequency) as LCR events detected in each cell normalized per 100 μm of the scanning line length and further normalized either per cycle or per 1 s of recording time. The Ca^2+^ signal of an individual LCR was estimated as follows: LCR = FWHMxFDHMx(F/F_0_-1)/2. The Ca^2+^ signal of the LCR ensemble within a cell was calculated as the sum of all Ca^2+^ signals of individual LCRs (ΔF/F_0_*μm*ms*#*2^−1^) and normalized per 100 μm of the line-scan image during a 1s time interval [21]. APCL was defined as the time intervals between the peaks of two adjacent AP-triggered Ca^2+^ transients. LCR period was defined as the time from the rapid upstroke of the prior AP induced Ca^2+^ transient to the onset of LCR [13]. Beat-to-beat APCL variability was assessed as the coefficient of variability (CV) calculated as standard deviation of APCL divided by its mean. All images were processed with IDL software (6.1., Research Systems).

### 2D Ca^2+^ signal measurements via a high-speed Hamamatsu camera

Ca^2+^ dynamics within isolated single SANC were measured by 2D imaging of fluorescence emitted by the Ca^2+^ indicator Fluo-4, using a high-speed, high resolution CCD camera (Hamamatsu C9100-12) as previously described [22]. We acquired data with the rate of 100 frames/second and spatial resolution of 512x160 pixels. The camera was mounted on a Zeiss Axiovert 100 inverted microscope (Carl Zeiss, Inc., Germany) with a x63 oil immersion lens and a fluorescence excitation light source (CoolLED pE-300-W, BioVision Technologies, Inc. PA, USA). We used only one emitter of blue light and only at 6% power setting. Fluo-4 fluorescence excitation (blue light, 470/40 nm) and emission light collection (green light, 525/50 nm) were performed using the Zeiss filter set 38 HE. Rhythmically beating cells were loaded with 3 μM Fluo-4AM for 10 minutes at room temperature. Fluo-4AM was subsequently washed out of the chamber, and Ca^2+^ signals were measured at 35±0.1°C at baseline and during BARs (1 μM ISO). To avoid phototoxicity, Fluo-4 was excited only for short periods of time (<10s). Data acquisition was performed using SimplePCI software (Hamamatsu Corporation, Japan). Scanline images were generated from 2D movies by our custom computer program (VAM).

## Drugs

Isoproterenol hydrochloride (C_11_H_17_NO_3_-HCl) (ISO) obtained from Sigma Aldrich.

## Statistics

Data are presented as mean ± SEM. The Student’s paired or unpaired *t-* test was used. A value of P<0.05 was considered statistically significant.

## Results

### Confocal imaging of spontaneous diastolic LCRs in single rhythmically beating guinea-pig (GP) and rabbit (R) SANC under basal conditions

Fig 1 illustrates representative examples of confocal line-scan images of spontaneous, diastolic LCRs and AP-induced Ca^2+^ transient recorded in SANC of guinea-pig (GP) and rabbit (R) (Fig 1A and 1B). Fig 1C presents a schematic illustration of the accurate scan-line orientation along cell border. The AP-induced Ca^2+^ transient cycle length (taken as the intervals between the peaks of two adjacent AP-triggered Ca^2+^ transients), a faithful proxy of the AP cycle length (APCL) [23], was comparable in GP and R SANC (Fig 1A and 1B). We observed spontaneous diastolic LCRs, indicated by asterisks beneath sarcolemma in both R and GP SANC (Fig 1A and 1B). Each LCR was characterized by its period, measured as the time between the rapid upstroke of the prior AP-triggered Ca^2+^ transient and the onset of the LCR in diastole (Fig 1D).

**Fig 2:**
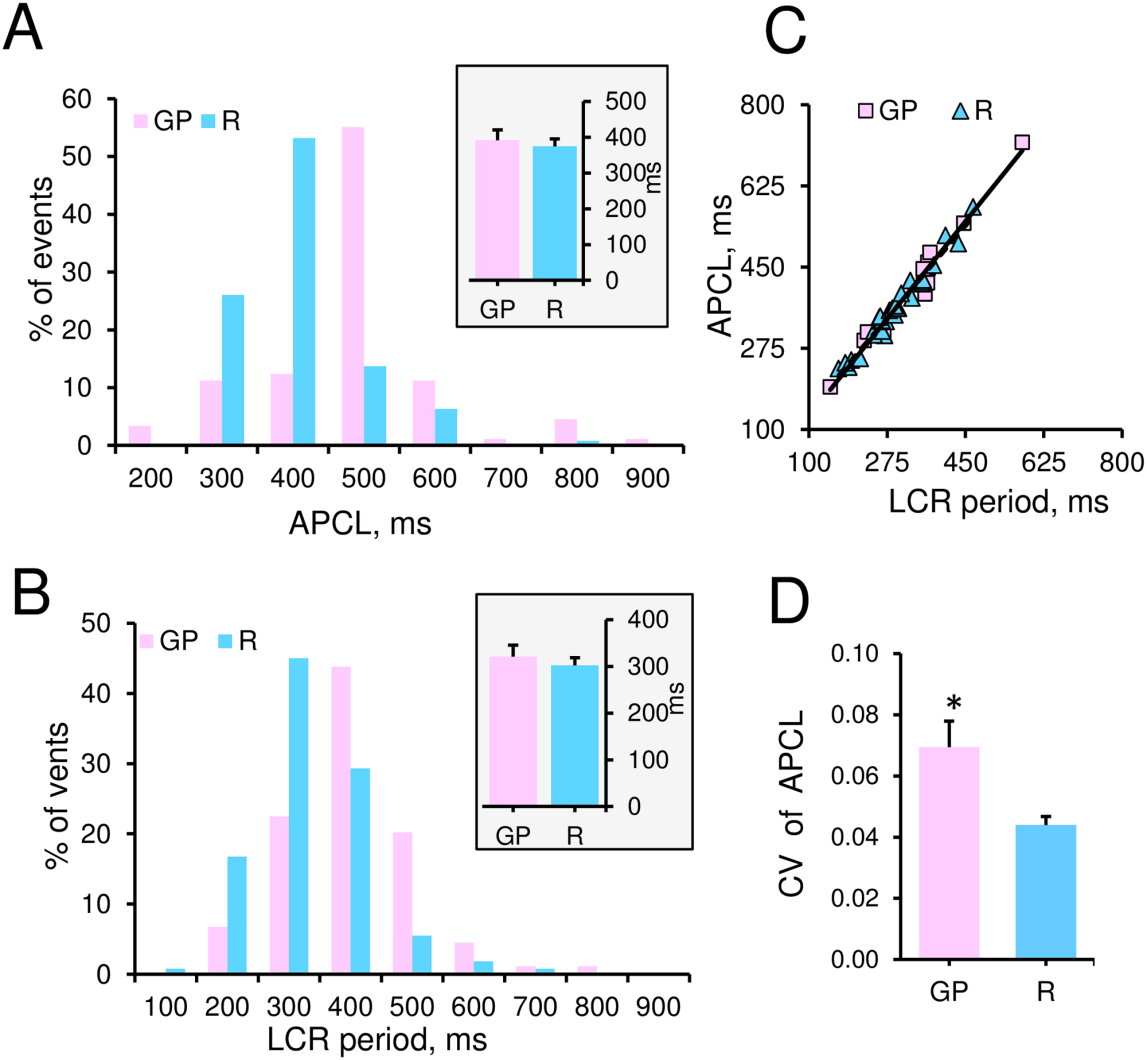
AP cycle length (APCL) in both GP and R single rhythmically beating SANC is tightly coupled to LCR period under the basal conditions. (A) Histogram of distributions of all individual APCLs, and (B) Individual LCRs periods in GP (91, LCRs from 13 cells) and R SANC (422 LCRs from 31 cells). Insets in (A) and (B) show the average APCL (interval between AP-induced Ca^2+^ transients) and LCR period (the time between the rapid upstroke of the prior AP-triggered Ca^2+^ transient and the onset of a LCR in diastole) in GP (n = 13) and R (n = 31) SANC. (C) Relationship of the average APCL to the average LCR period is subtended by the same linear function in GP SANC (y = 1.2x + 8.3; R^2^ = 1.0; n = 13) and R SANC (y = 1.1x + 27.8; R^2^ = 1.0; n = 31). (D) Average CV of APCL (measured as SD/Mean) in GP and R SANC.

Distributions of all APCLs and all LCRs period measured by confocal microscopy in spontaneously beating GP and R SANC exhibited a similar pattern, but was slightly skewed towards larger APCL values in GP SANC (Fig 2A and 2B). The average APCL and LCR period, and their relationship were comparable in both GP and R SANC (Fig 2 insets). Specifically, the average APCL was 392.0±28.7 ms in GP SANC, and in R SANC this parameter was slightly, but not significantly smaller 374.5±20.9 ms (Fig 2A inset). The average LCR period of GP SANC was 321.1±24.4 ms compared to 302.0±16.4 ms in R SANC (Fig 2B inset). Relationship of the average APCL to the average LCR period was subtended by the same linear function in both, GP and R SANC (Fig 2C). The average beat-to-beat APCL variability (calculated as CV, see Methods) was larger in GP SANC (0.07±0.01, n = 13) vs that in R SANC (0.04±0.003, n = 31, *P<0.05) (Fig 2D).

**Fig 3:**
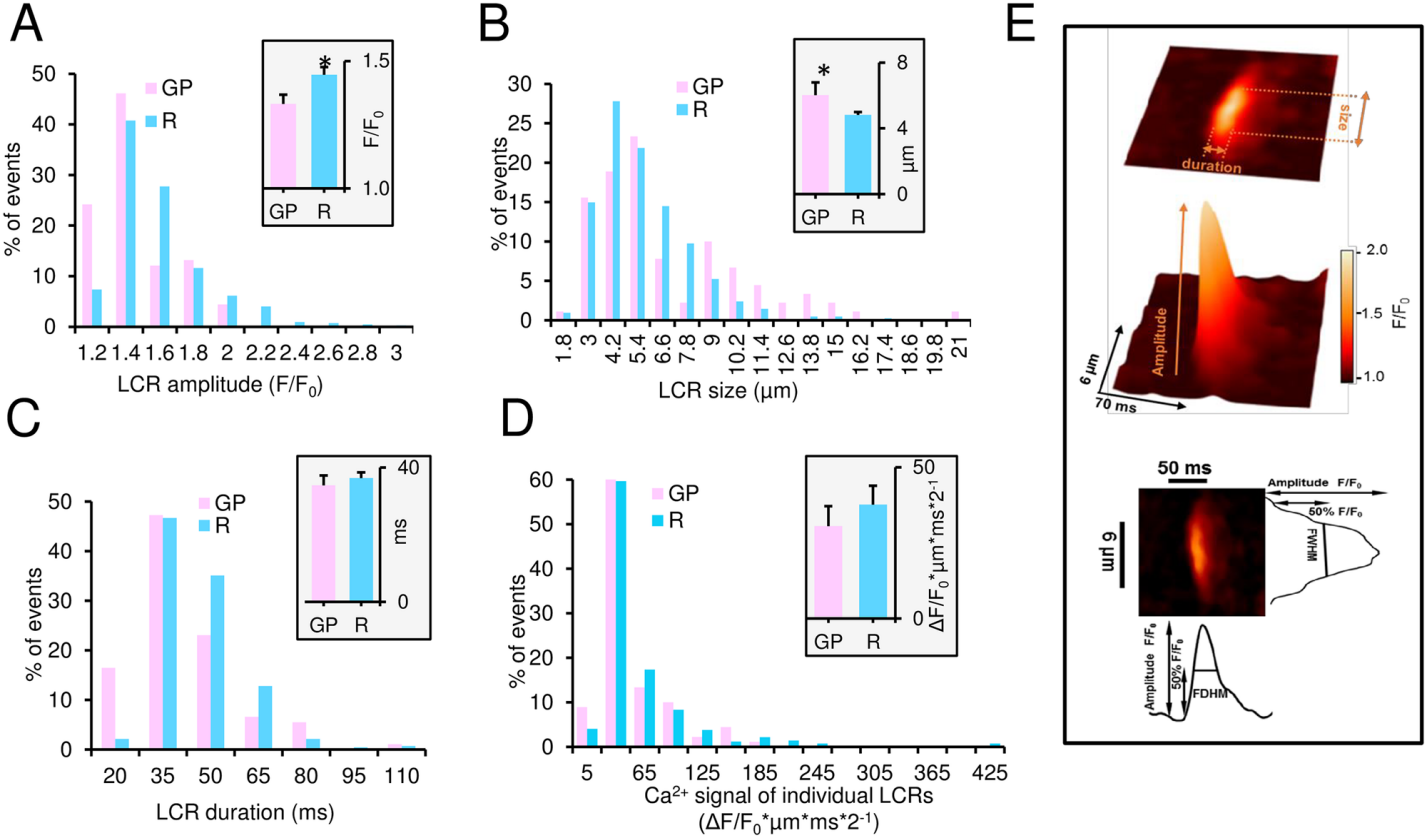
Distributions and average LCR characteristics measured by confocal microscopy in single spontaneously beating GP and R SANC under the basal conditions. Histogram distribution of LCR characteristics: (A) amplitude (as normalized Ca^2+^ fluorescence, F/F_0_); (B) size (μm); (C) duration (ms); (D) Ca^2+^ signals of individual LCRs (ΔF/F_0_*μm*ms*2^−1^) (see Methods and Panel E) in GP (91, LCRs from 13 cells) and R SANC (422 LCRs from 31 cells). Insets (A-D) show the average data in GP (n = 13 cells) and R SANC (n = 31 cells). *P<0.05 by unpaired *t*-test. (E) An example of two-dimensional and three-dimensional confocal line-scan images and surface plot of an LCR, demonstrating our measurements of LCR characteristics.

**Fig 4:**
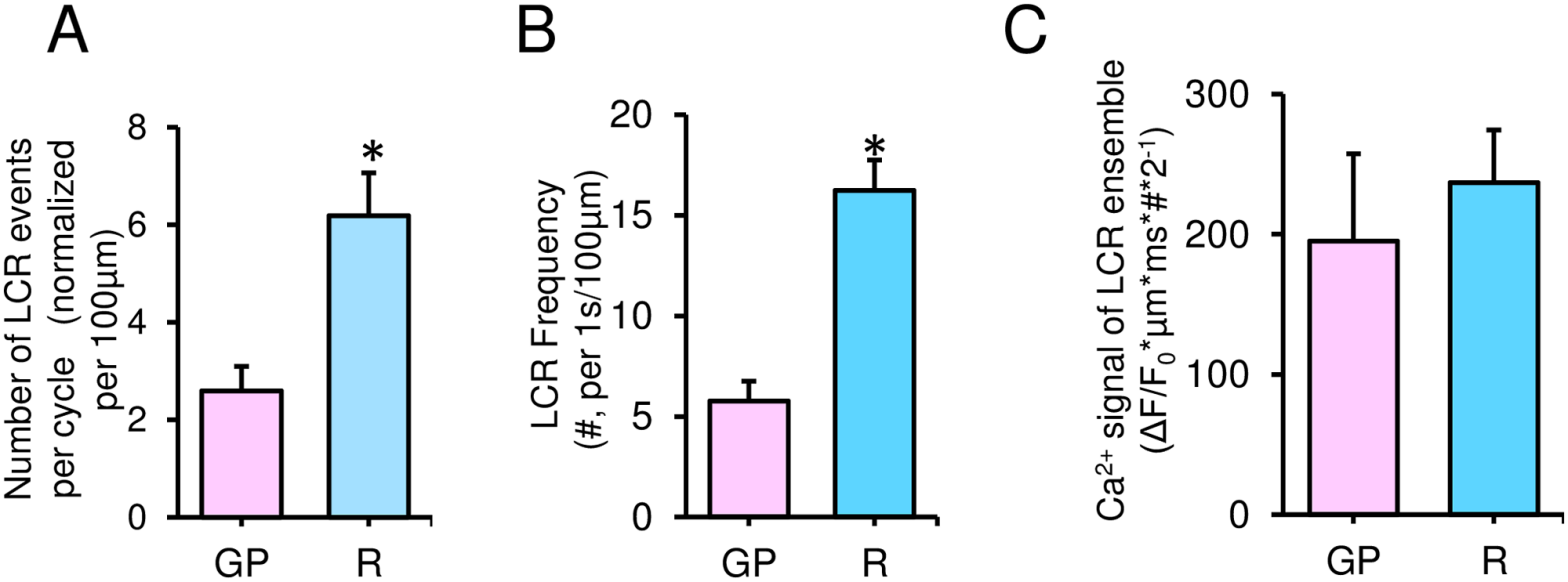
Number of LCR events and magnitude of Ca^2+^ signals of the LCR ensemble in single rhythmically beating GP and R SANC. (A) The average number of LCR per cycle, normalized to 100 μm of scanning line in each cell. (B) The average number of LCR, normalized per 100 μm of the line-scan image and per 1-s time interval of recording. (C) Average amplitudes of Ca^2+^ signals of the LCR ensemble (as the integrated Ca^2+^ signal produced by each LCR ((ΔF/F_0_*μm*ms*#*2^−1^), see Methods), normalized per 100 μm of the line-scan image during a 1-s time interval in GP (n = 13 cells) and R SANC (n = 31 cells). *P<0.05 by unpaired *t*-test.

We obtained additional insights about other LCR characteristics (amplitude, size, duration and amplitude of the Ca^2+^ signal of individual LCRs, see Methods and Fig 3E) in GP and R SANC by examining and comparing both distributions and the average of those parameters at baseline conditions (Fig 3). The average LCR amplitude (Fig 3A inset) was lower in GP SANC (1.3±0.03 F/F_0_ versus 1.5±0.03 F/F_0_ in R SANC), due to the presence of substantial number of small amplitude LCRs (within the first 1.2 bin, Fig 3A). The average LCR size, however, (Fig 3B inset) was larger in guinea pig SANC (6.1±0.7 μm versus 4.8±0.2 μm in R SANC), due to the presence of LCRs larger than 10 μm (Fig 3B). The average LCR duration (Fig 3C inset) and individual LCR Ca^2+^ signal (Fig 3D inset) were almost the same in both species, even though the distributions of both parameters were slightly more spread toward bigger numbers in R SANC. The average LCR frequency reported as the number of LCR either per cycle (Fig 4A) or per 1 s (Fig 4B) (see Methods) was significantly lower in GP vs that in R SANC (2.6±0.5 events vs. 6.2±0.9 events in Fig 4A and 5.7±0.9 events vs. versus 16.2±1.5 events in Fig 4B). The average amplitude of normalized Ca^2+^ signal of the LCRs ensemble (Fig 4C), which represents the product of all LCR characteristics calculated for each cell (ΔF/F_0_*μm*ms*#*2^−1^, see Methods) was comparable in GP (194.9±62.5 ΔF/F_0_*μm*ms*#*2^−1^) and R SANC (236.8±37.5 ΔF/F_0_*μm*ms*#*2^−1^).

### β-AR stimulation-induced (BARs) changes in LCRs measured by confocal microscopy in single rhythmically beating GP

**Fig 5:**
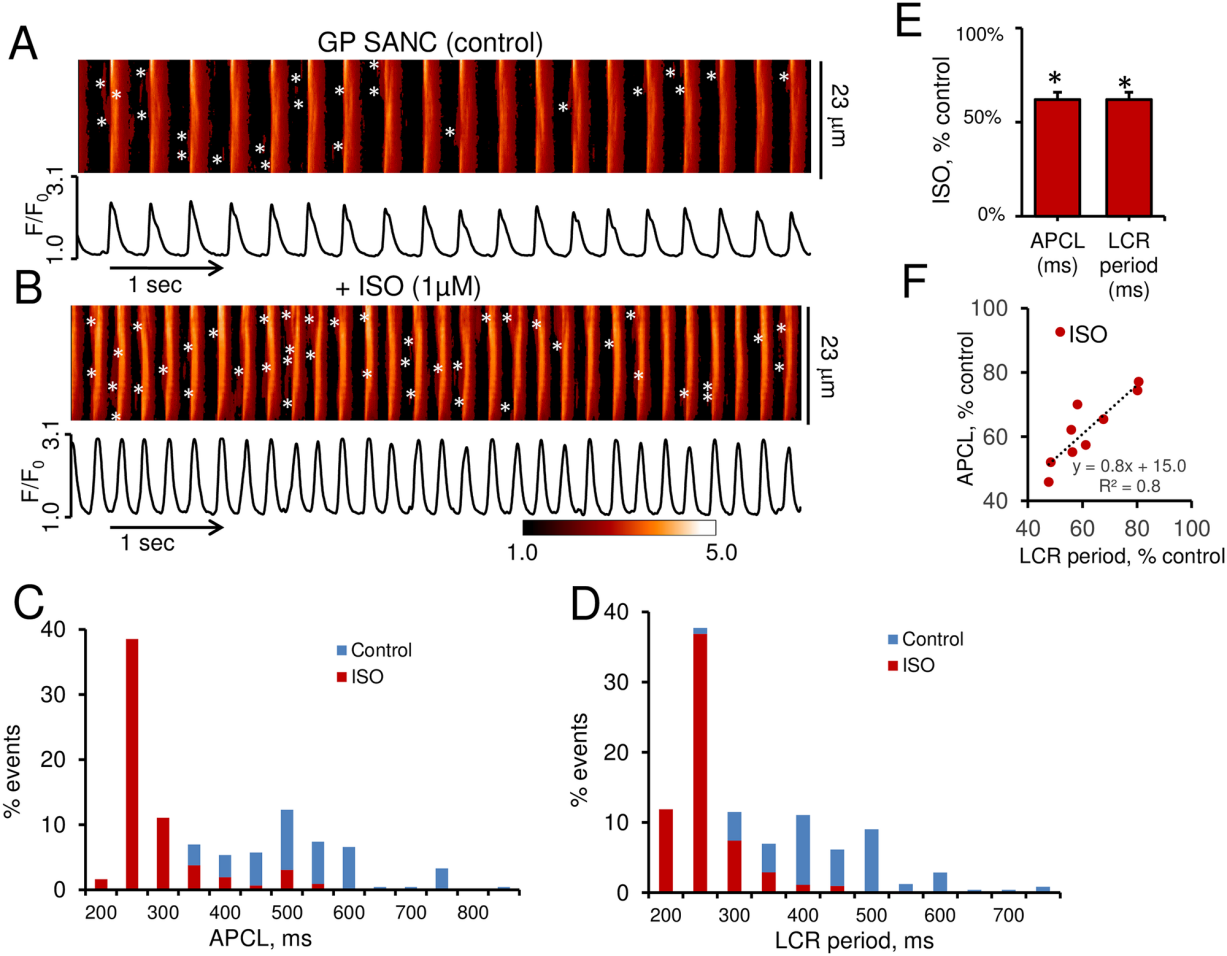
β-AR stimulations (BARs) decreases the AP-induced Ca^2+^ transient cycle lengths (APCL) and LCR periods in single spontaneously beating GP SANC. Representative examples of confocal line-scan images (upper panels) and AP-induced Ca^2+^ transients (lower panels) of GP SANC prior to (A) and in response to 1 μM of Isoproterenol (ISO) in the same cell (B). LCRs are marked with asterisks. BARs (1 μM ISO) shifts the distributions of APCL (C) and LCR period (D) to the left in GP SANC. (E) The average change of APCL and LCR period following ISO exposure (% of control) (n = 9, *P<0.05 versus control by paired *t*-test). (F) Relationship of the average APCL (% of control) to the average LCR period change (% of control) in response to ISO in GP SANC (n = 9).

BARs in R SANC is accompanied by reduction in LCR period and APCL and an increase in LCR size [12]. Fig 5A and 5B illustrate representative examples of confocal line-scan images (upper panel) and Ca^2+^ transients (lower panel) in GP SANC prior (Fig 5A) and in response to BARs by ISO (1 μM) (Fig 5B). In GP SANC, ISO (Fig 5B) notably increased spontaneous firing rate compared to that at baseline. The average spontaneous APCL was significantly reduced by 38.0% during BARs from 486.3±37.2 ms at baseline to 301.2±25.7 ms, and the average LCR period by 38.2% from 411.3±26.3 ms at baseline to 249.9±15.5 ms following BARs (Fig 5E). Fig 5C, 5D and 5F indicate that BARs shifts the distributions and relative relationships of APCLs and LCR periods toward shorter APCLs and LCR periods as previously reported in R SANC [12].

**Fig 6:**
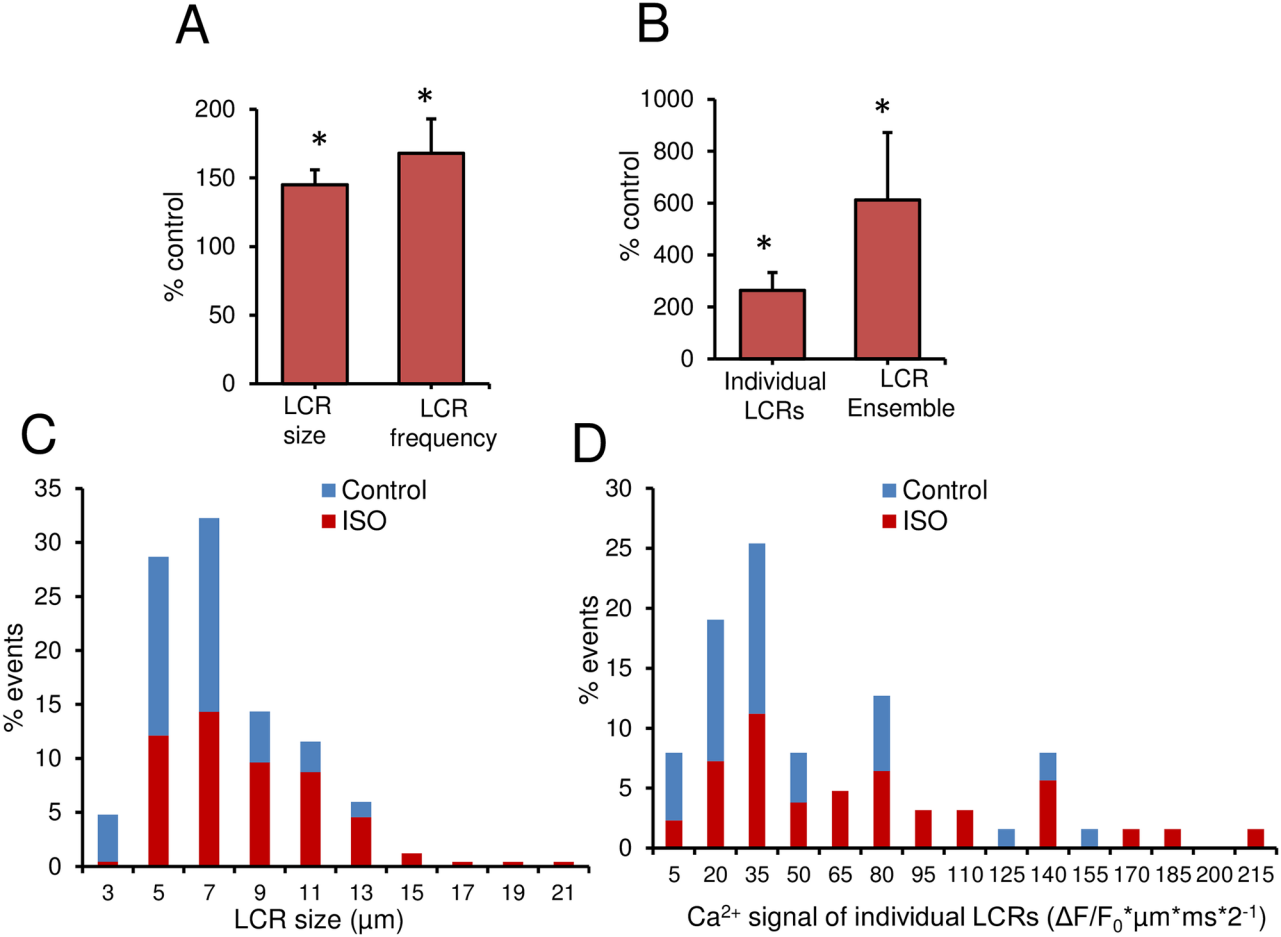
Effect of β-AR stimulations (BARs) on average and distributions of LCR characteristics in spontaneously beating GP SANC. BARs (ISO, 1 μM) increase (A) the average LCR size (μm) and LCR frequency (#, normalized per 100 μm and per 1 s) in GP SANC and (B) the average amplitude of the Ca^2+^ signal of individual LCRs (ΔF/F_0_*μm*ms*2^−1^) and Ca^2+^ signal of the LCR ensemble (ΔF/F_0_*μm*ms*#*2^−1^). *P<0.05 versus control by paired *t*-test (n = 9). Distributions of LCR sizes (C) and Ca^2+^ signals of individual LCRs (D) are shifted in GP SANC toward higher values in response to 1 μM of ISO (84 LCRs in control and 168 LCRs with ISO from n = 9 cells).

BARs also significantly increased LCR size by 44.7% (from 5.2±0.4 μm to 7.4±0.7 μm) and LCR rate by more than 50.0% (from 6.9±1.1 events per 100μm per 1 s to 10.5±1.6 events per 100μm and per 1s) (Fig 6A). The LCR duration and LCR amplitude tended to increase but did not reach statistical significance (data not shown). The average Ca^2+^ signal of individual LCRs and the Ca^2+^ signal of the LCR ensemble significantly increased by 2- and 3-fold, respectively, in response to BARs (Fig 6B). Histogram profiles of selected LCR parameters before and during BARs are illustrated in Fig 6C and 6D. Note that ISO (1 μM) shifted LCR parameters distributions toward higher values. Therefore, BARs modulates beating rate in GP SANC exactly like its effect in R SANC [12, 24].

### 2D calcium signals dynamic measured by high-speed imaging system in single rhythmically beating GP SANC

**Fig 7:**
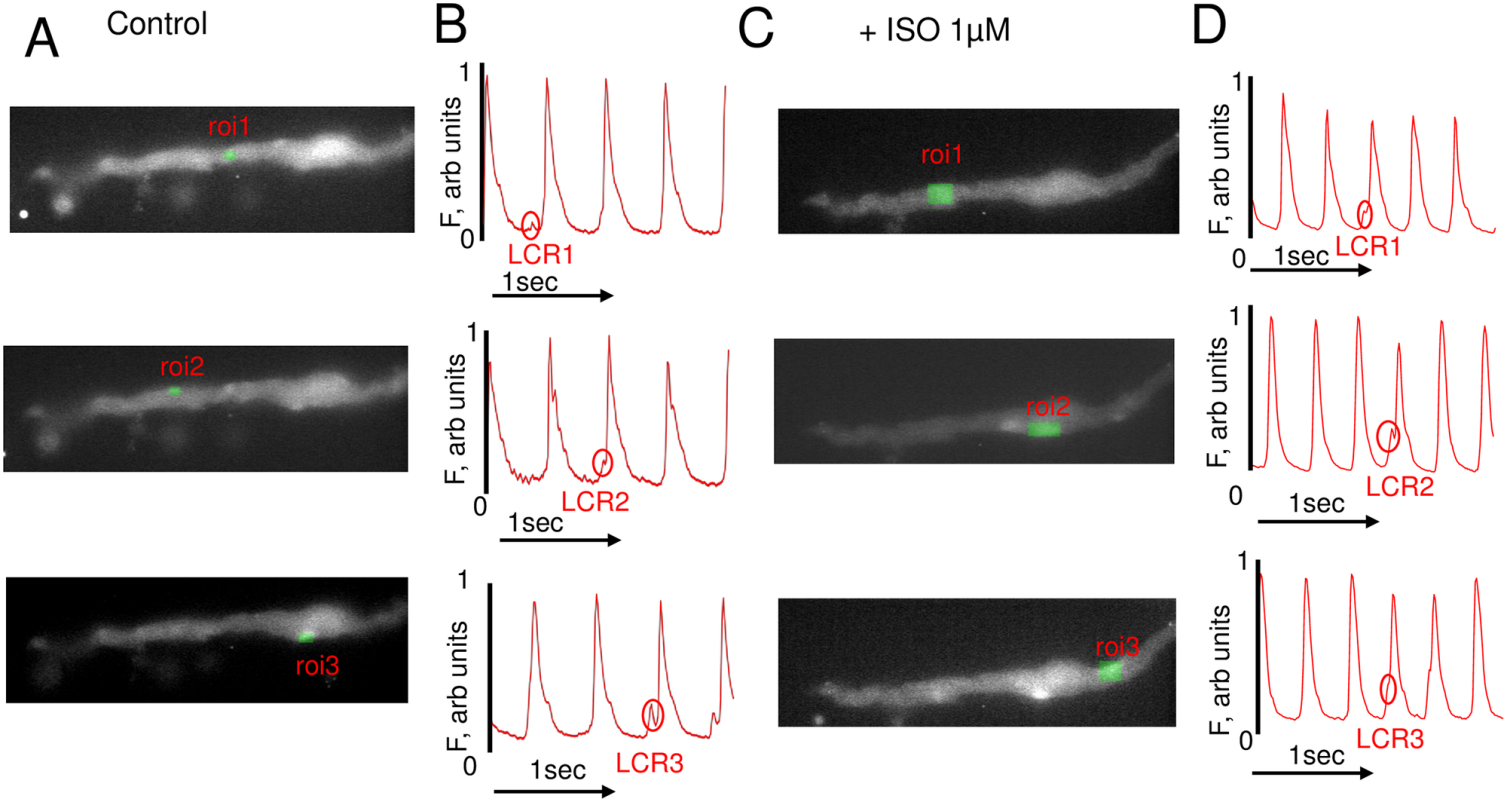
Spontaneous diastolic LCRs detected by high-speed 2D imaging under the basal conditions and in response to β-AR receptor stimulations (BARs). (A) Ca^2+^ images under control and (C) during 1 μM of ISO measured by the high-speed 2D camera. The three green rectangles, region of interest (ROI) in (A) and (C) represent arbitrary locations within the cell perimeter to illustrate spontaneous diastolic LCRs. (B) and (D). Respective time series of the of AP-induced Ca^2+^ transients and spontaneous diastolic LCRs (a small bump preceding AP-induced Ca^2+^ transient and indicated by oval), measured within ROI 1–3 in (A) and (C) in control conditions and during superfusion with 1 μM of ISO.

To illustrate presence of LCRs in the entire cell, and not only over a relatively small area of the cell along a scan-line we recorded LCRs in 2D with a high-speed 2D Hamamatsu camera in three spontaneously beating GP SANC (illustration in Fig 7 and S1–S6 Movies) in control conditions (see S1, S3 and S5 Movies) and during superfusion with ISO (1 μM) (see S2, S4 and S6 Movies). Fig 7A and 7C, and movies (S1 and S2 Movies) show the Ca^2+^ signal recorded with a high-speed 2D camera in the same GP SANC in control (S1 Movie) and during ISO (S2 Movie). While LCRs are clearly observed in our movies, we also illustrated the emergence of LCRs over time by plotting the Ca^2+^ signal in three different locations within the cell (ROI1, ROI2, ROI3, green rectangles in panels A and C) where LCRs were present (marked by circles in panels B and D). Like in R SANC, majority of LCRs in GP SANC were located along the periphery (S1–S6 Movies). It is evident that in response to BARs by ISO, spontaneous diastolic LCRs become more frequent (S2, S4 and S6 Movies) and occur at reduced period, and the AP-induced Ca^2+^ transients follow these LCRs occurred with reduced cycle length, resulting in an increase of beating rate that is consistent with our confocal line-scanning results (Fig 5).

We further illustrated importance of proper selection of scanline for successful recording of LCRs by comparing LCRs recorded by Hamamatsu camera (S3 Movie) in GP SANC and LCRs generated by a computer program within two virtual scanlines (Scanline 1 and 2 in S1 Fig) using the same data. We found that during 7 pacemaker cycles, the amplitudes of AP-induced Ca^2+^ transients were similar within both virtual scanlines (superimposed green and red Ca^2+^ signals in S1 Fig). However, the number of LCRs, generated along scanline 1 was significantly smaller than that along scanline 2 (LCRs indicated by arrows).

Next, we studied relationship between the total number of LCRs per cycle along the virtual scanline 2 (S1 Fig) and the total number of LCRs in the movie (S3 Movie) in each cycle measured as previously described [22]. We found that these two parameters correlate with R^2^ = 0.74 (S1 Fig). However, both LCRs in 2D and in the scanlines exhibited substantial beat-to-beat variations due to stochastic nature of LCR generation by RyRs. The scanline reported about 36% of all LCR in 2D (35 total LCRs in virtual scanline vs 97 LCRs in 2D). Thus, the fluctuating LCRs that did not occur on the scanline weaken the correlation.

## Discussion

Since this is the first study of LCRs in GP, our first finding was that GP SANC indeed generate diastolic LCRs in spontaneously firing SANC similar to those in R SANC as reported previously and reported here under the same experimental conditions (Figs 1–4). Specifically, we show, for the first time, that both LCRs and AP-induced Ca^2+^ transients in spontaneously beating GP SANC are comparable to those in R SANC.

LCRs of SANC in both species are generated beneath sarcolemma. All studied cells in both species exhibited LCRs. Previous studies, however, did not detect the LCR in GP using confocal line-scan microscopy or Nipkow disk techniques [19]. This apparent discrepancy with our results can be explained on the technical grounds. Our results indicate that detection of the LCRs requires accurate positioning of the scanning line, directly near sarcolemma, where the clusters of RyR release channels reside in SANC (see Fig 1 in [25]). It is also important to note that not every location beneath the sarcolemma generates LCRs. Furthermore, due to their stochastic nature of activation, individual LCRs have different characteristics during each cycle as evident from our 2D images and movies. Thus, to detect LCRs, the scanning line should not be set randomly, but rather along specific locations of LCR preferable activity over several cycles that have been prescreened with x-y confocal imaging (see Methods and S1 Fig). Further, many LCRs during late diastole merge into AP-induced Ca^2+^ transient, and should not be confused with the Ca^2+^ transient onset per se. Small LCRs have reduced amplitudes and durations, and require careful contrast and balanced laser power for Ca^2+^ indicator excitation.

We further found that the average LCRs amplitude, number LCR per cycle and LCR frequency in GP versus R SANC are smaller (Figs 3A and 4A and 4B), but the LCR size in GP versus R SANC is larger by 20% (Fig 3B). The LCR duration and the average Ca^2+^ signal of individual LCRs do not differ in both species (Fig 3C and 3D). Further, the amplitude of Ca^2+^ signal of the LCR ensemble, a crucial LCR parameter, which determines the inward NCX current during diastole and therefore contributes in the regulation of APCL, is comparable in GP and R SANC. Both cell types exhibit tight couplings between LCR period and APCL, and this coupling is subtended by the same linear function in SANC isolated from both species [21, 26–28]. The fact that LCRs in both GP and R SANC contribute to the diastolic depolarization and regulation of APCL indicate that coupled-clock functions involved in pacemaker function are not species limited. Finally, GP SANC exhibit similar LCRs and APCL responses to BARs, as shown previously for LCRs of R SANC [12]. Specifically, BARs concurrently decreases the average LCR period and APCL, but increases the average LCR size, number of LCR events, Ca^2+^ signals of individual LCRs, and Ca^2+^ signal of the LCR ensemble in SANC of both species types.

We have recently shown that beat-to-beat fluctuations in the average period of LCRs are linked to intrinsic APCL variability [22]. A more recent study by Zaniboni et al. [29] has compared membrane and Ca^2+^ clocks with regard to pacing rate variability in spontaneously beating GP SANC, suggesting a primary role of LCRs in determining beat-to-beat ACL variability (and a stabilizing effect of the membrane clock). In the present study, we found that beat-to-beat CL variability measured as coefficient of variation (CV = SD/mean) is higher in guinea pig SANC, whereas average firing rate is not different in GP and R SANC. This can be explained by our other finding that the number of LCR events is substantially smaller in GP (Fig 4). Indeed, each LCRs is a stochastic event, and its impact on the APCL is expected to be grater when the number of LCRs is smaller (and the role and impact of each LCR within the LCR ensemble signal is substantial). These results provide additional evidence for key importance of LCRs for beat-to-beat APCL variability.

In summary, while the LCRs in both species slightly differ (e.g. in size), their presence and tight coupling to APCL support the idea of their importance as a **general** pacemaker mechanism within a coupled-clock system in mammalian species. Furthermore, the flight-or-fight reflex is also accomplished via the same general mechanism, i.e. shifts in LCR characteristics coupled to AP firing rate from their basal state values (Fig 2C). In response to BARs, LCRs occur earlier in time during diastolic depolarization and are larger in size, resulting from their synchronization, generating a LCR ensemble Ca^2+^ signal (Figs 5–7) that would be expected to generate an earlier and larger NCX inward current, accelerating the AP firing rate [21]. While our paper is focused on biophysical detection and examination of LCRs and their link to rate regulation, other important aspects of local signaling, including biochemical signaling (especially phosphorylation) also merit further investigation in different species in the context of the idea of a coupled-cock system as a general pacemaker mechanism for all mammalian species.

## Study limitations

While we report CV of APCL, our results should be treated with caution, because our AP-induced Ca^2+^ imaging recordings were limited in time to avoid cell photodamage and bleaching.

## Supporting information

S1 Fig Importance of proper setting of scanline for successful detection of LCRs illustrated by the relation of LCRs recorded by 2D microscopy and LCRs detected in scanlines using the same data shown in S3 Movie. (A, B) The panels show placement of 2 virtual scanlines in the cell area. (C, D) Respective scanline images generated by our custom computer program report substantially different LCR activity. LCRs are shown by small yellow arrows. (E) Time series of Ca^2+^ signals generated within 3.3 μm bands within the two virtual scanlines about in the middle of the linescan images marked by green and red thick lines and respective dotted line boxes in C and D. The overlapped time series indicate that while LCR activity is substantially different in the images, the AP-induced Ca^2+^ transient has almost the same peak amplitude, indicating that LCRs indeed absent along scanline 1, rather than missing due to a low indicator signal (LCRs marked by black arrows). (F) Relation of LCR numbers in each cycle (labeled by white numbers in panel D) reported by the scanline 2 and detected in 2D. The respective trend line and correlation coefficient (R^2^) are shown on the plot.
(TIF)

S1 Movie An example of Ca^2+^ signal dynamics (including LCRs) recorded by a high-speed camera in a guinea-pig SA node cell under control conditions (same cell as in Fig 7). (WMV)

S2 Movie The same cell as in S1 Movie (and in Fig 7) but in the presence of beta-adrenergic receptor stimulation (during superfusion with 1 μM of ISO). (WMV)

S3 Movie Ca^2+^ signal dynamics in a second example of guinea-pig SA node cell under control conditions. (WMV)

S4 Movie Ca^2+^ signal dynamics in the same cell as in S3 Movie, but in the presence of ISO (1 μM). (WMV)

S5 Movie Ca^2+^ signal dynamics in a third example of guinea-pig SA node cell under control conditions. (WMV)

S6 Movie The same cell as in S5 Movie, but in the presence of ISO (1 μM). (WMV)
