# Supplementary figures and images for: Spontaneous, local diastolic subsarcolemmal calcium releases in single, isolated guinea-pig sinoatrial nodal cells
Source: PLoS One. 2017 Sep 25;12(9):e0185222. doi: 10.1371/journal.pone.0185222 (PMC5612473; doi:10.1371/journal.pone.0185222)

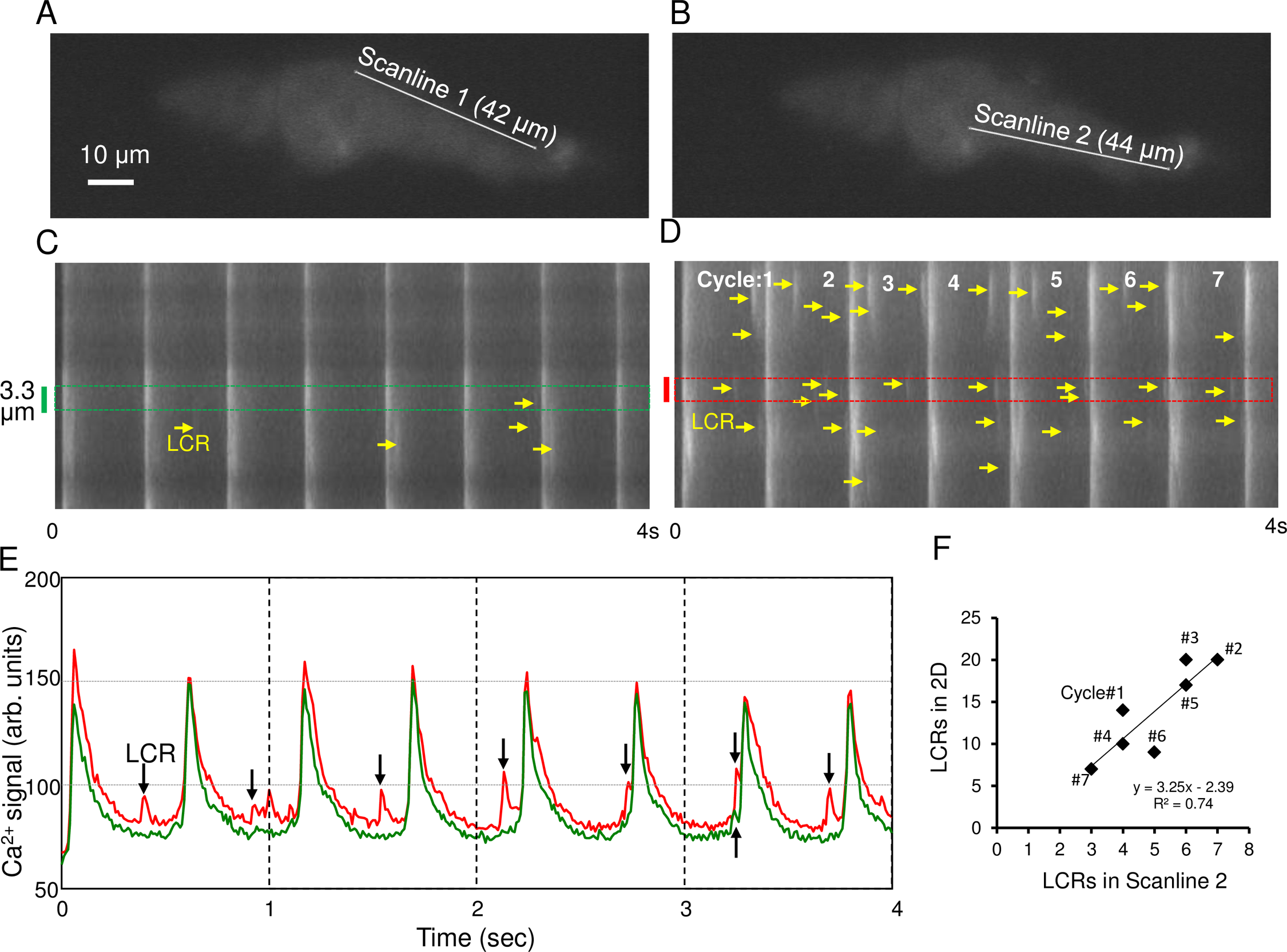

Supplement: S1 Fig — (A, B) The panels show placement of 2 virtual scanlines in the cell area. (C, D) Respective scanline images generated by our custom computer program report substantially different LCR activity. LCRs are shown by small yellow arrows. (E) Time series of Ca2+ signals generated within 3.3 μm bands within the two virtual scanlines about in the middle of the linescan images marked by green and red thick lines and respective dotted line boxes in C and D. The overlapped time series indicate that while LCR activity is substantially different in the images, the AP-induced Ca2+ transient has almost the same peak amplitude, indicating that LCRs indeed absent along scanline 1, rather than missing due to a low indicator signal (LCRs marked by black arrows). (F) Relation of LCR numbers in each cycle (labeled by white numbers in panel D) reported by the scanline 2 and detected in 2D. The respective trend line and correlation coefficient (R2) are shown on the plot. (TIF) [file pone.0185222.s001.tif]
